# Multidimensional Approach of Genotype and Phenotype in Stroke Etiology: The MAGPIE Study

**DOI:** 10.1002/hsr2.70227

**Published:** 2024-12-04

**Authors:** Redoy Ranjan, Dipannita Adhikary, Shanto Barman, Md. Shuktarul Islam, Gie Ken‐Dror, Md. Abdullah Yusuf, Adneen Moureen, Maliha Hakim, Pankaj Sharma

**Affiliations:** ^1^ Department of Biological Sciences Royal Holloway University of London London UK; ^2^ Department of Cardiac Surgery Bangabandhu Sheikh Mujib Medical University Dhaka Bangladesh; ^3^ Department of Medicine Mugda Medical College & Hospital Dhaka Bangladesh; ^4^ Department of Neurology National Institute of Neurosciences and Hospital Dhaka Bangladesh; ^5^ Department of Microbiology National Institute of Neurosciences and Hospital Dhaka Bangladesh; ^6^ TB New Technologies and Diagnostics The United States Agency for International Development (USAID) Dhaka Bangladesh; ^7^ Department of Clinical Neuroscience Imperial College Healthcare NHS Trust London UK

**Keywords:** Bangladesh, etiology, genetic study, hemorrhagic stroke, ischemic stroke, stroke

## Abstract

**Background and Aims:**

Stroke is a leading cause of mortality and morbidity in Bangladesh. It is estimated that genetic determinants account for around 40%–60% of its etiology, similar to environmental factors. This study aimed to provide a better understanding of the genetic, environmental, and clinical risk factors in stroke patients from Bangladesh.

**Methods:**

The MAGPIE (Multidimensional Approach of Genotype and Phenotype In Stroke Etiology) study is a population‐based case‐control study that will allow a hypothesis‐free genome‐wide association study (GWAS) to identify genetic risk factors associated with adult stroke (age ≥ 18 years) in Bangladesh. This study will collect detailed phenotypic data as well as blood samples from stroke patients and control subjects. High‐molecular‐weight genomic DNA will be isolated and archived using Qiagen DNA isolation kits.

**Results:**

We will utilize SPSS v28.0, vR‐4.3.2 and gPLINK v2.0 software to analyse the study variables, as appropriate. Further, appropriate statistical tests will be applied to test the significance level between study groups. As applicable, data will be presented in tables and graphs, such as Manhattan plots and Quantile‐Quantile (QQ) plots. A *p* < 0.05 will be considered as statistical significance.

**Conclusion:**

This will be the first large‐scale carefully phenotyped biobank of Bangladeshi stroke patients which will enable a GWAS enabling an understanding of the association between gene‐phenotype risk factors which has the potential to revise and refine national stroke guidelines.

## 1 Introduction

Stroke is a significant health concern in Bangladesh, with a growing incidence and prevalence [1, 2, 3]. Approximately 217 per 100,000 people suffer a stroke annually, a figure that is expected to significantly rise due to an aging population and increasing prevalence of risk factors such as hypertension and diabetes [2, 3, 4, 5]. Stroke is a leading cause of mortality in Bangladesh, accounting for about 9% of all deaths. The high fatality rate is attributed to limited access to healthcare, inadequate stroke management facilities, and a lack of awareness about stroke symptoms and prevention [5, 6, 7]. Further, South Asians comprise 20% of the world's population, while those of European descent account for 13% [8, 9, 10]. Due to the genetically stratified structure of ancestral populations, disease genetics are believed to vary across different ethnic groups. Population‐based studies reveal significant differences in stroke incidence among ethnic groups that environmental factors cannot fully explain [10]. Several modifiable risk factors, including age, sex, hypertension, diabetes, and prior diseases, contribute to these differences. Addressing these challenges by understanding genetic factors becomes crucial to reducing the stroke burden in Bangladesh as well as in South Asia, which is expected to rise significantly over the next decade [11, 12].

Diagnosing a stroke can be challenging and further complicated by the lack of reliable biomarkers or genes to predict risk [11, 12, 13, 14, 15]. However, well established risk factors alone cannot explain why some individuals are more susceptible to environmental determinants than others. The offspring of stroke patients have been found to have a fourfold increase in the risk of stroke, and there is evidence of ischemic stroke clustering in families [16, 17, 18, 19, 20]. Further, genetic factors may contribute to commonly acquired strokes and rare familial stroke syndromes, especially sickle cell disease and cerebral autosomal dominant arteriopathy with subcortical infarcts and leukoencephalopathy, which are more prevalent in certain races [21, 22].

In recent literature, candidate‐gene case‐control studies have identified multiple but consistent genetic associations with stroke, such as factor V Leiden, methylenetetrahydrofolate reductase (MTHFR), prothrombin, and angiotensin‐converting enzyme (ACE) [11, 12, 20, 21, 22, 23, 24]. However, some are risk factors for one stroke subtype but may be protective against another; for example, factor V Leiden is a risk factor for ischemic stroke but likely protective against hemorrhagic stroke [25]. Studies have also suggested that MTHFR, ACE, and Apolipoprotein E (ApoE) genes are risk factors for hemorrhagic stroke in European Caucasians, while factor V Leiden has protective rules [24, 25, 26]. Furthermore, these genes have also been associated with a higher risk of ischemic stroke among Asian descent, specifically Chinese, Korean and Japanese populations. The BRAINS (Bio‐repository of DNA in stroke) consortium [27, 28] has also found Factor V Leiden, MTHFR, and Prothrombin as genetic risk factors for stroke among South Asians and offers hope for developing population‐specific drugs.

The primary objective of the MAGPIE study is to develop a comprehensive understanding of the clinical and genetic risk factors contributing to stroke among the Bangladeshi population. A secondary objective is to collaborate with the international BRAINS study [27, 28] to compare this population with other South Asian migrant populations against those of Caucasian ancestry.

## 2 Research Gap: The Rationale of the Study

Stroke is a leading cause of death in Bangladesh with increasing morbidity and mortality. Although Genome‐wide association studies (GWAS) have implicated multiple loci in ischemic stroke, the biological mechanism of the causal associations between the relevant genes and stroke remains uncertain especially in South Asians. Although genetic studies on stroke patients are well‐established in Western countries such as Europe, the UK, and United States, we have yet to conduct a single GWAS study in Bangladesh. Stroke has a genetic basis, and identifying the genes involved may help us define the mechanisms that cause the disease and identify novel therapeutic targets to improve life expectancy among the Bangladeshi population.

## 3 Study Objectives

Our primary goal is to identify gene loci associated with different types of stroke through genetic analysis among the Bangladeshi population. In addition, we aimed to develop a DNA repository of well‐characterized stroke patients in Bangladesh. Furthermore, the study will also investigate the association of gene variations with stroke subtypes, ethnic groups, and clinical risk factors in collaboration with the international BRAINS study.

## 4 Study Methodology

### 4.1 The MAGPIE Study

**Figure 1:**
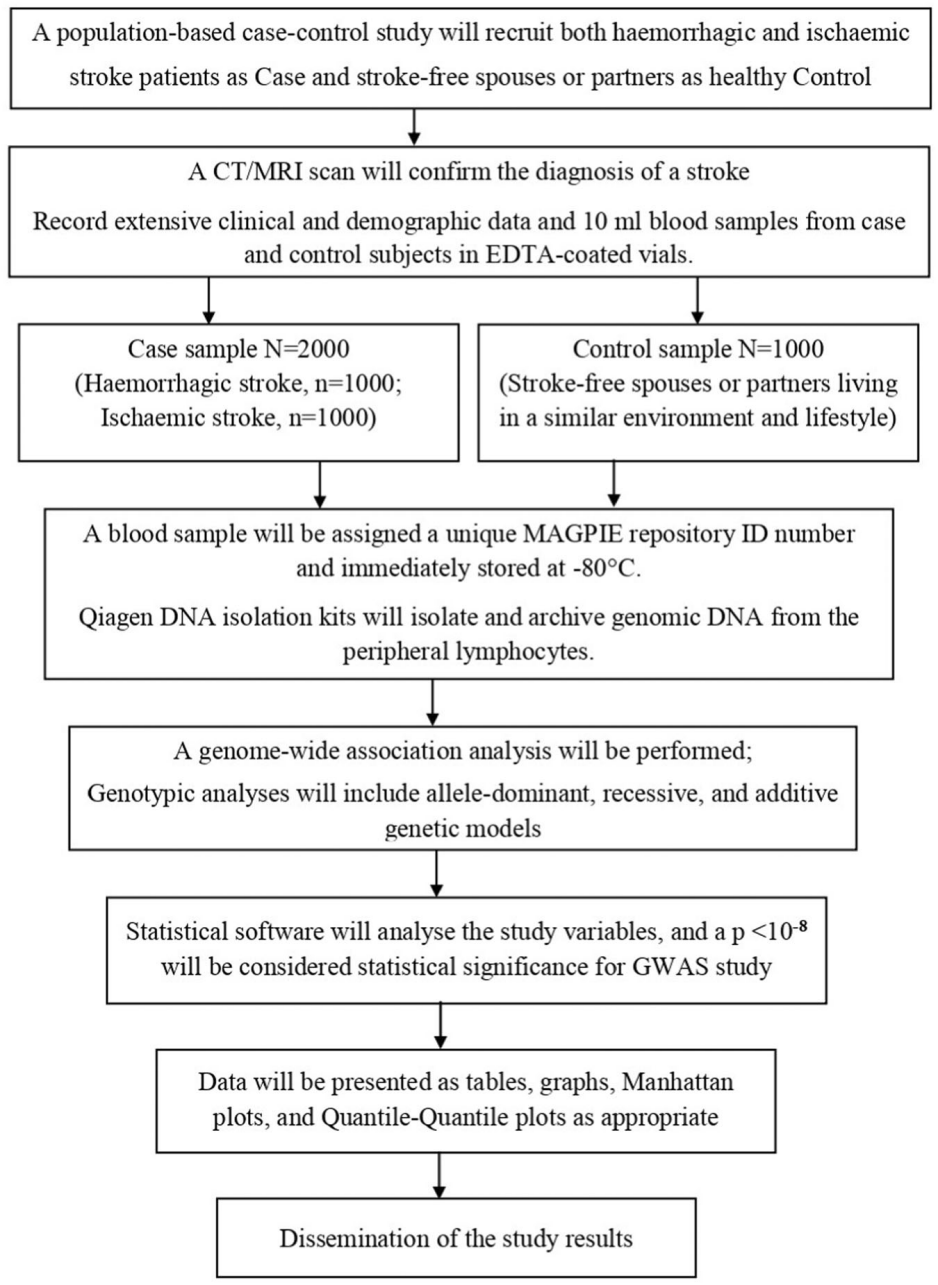
The comprehensive flowchart of the MAGPIE study. CT, computed tomography; GWAS, genome‐wide association study; MAGPIE, **M**ultidimensional **A**pproach of **G**enotype and **P**henotype **I**n **S**troke **E**tiology; MRI, magnetic resonance imaging.

This is a population‐based case‐control study that will be conducted at the National Institute of Neuroscience and Hospital (NINS&H) and Bangabandhu Sheikh Mujib Medical University (BSMMU), Bangladesh. All type stroke patients as Case/Proband and their stroke‐free spouse or partner as healthy Control subjects will be recruited. Informed written consent will be taken from the study population. We aim to recruit 3000 subjects, of whom 2000 will be cases (Group 1; 1000 patients for each hemorrhagic and ischemic stroke group) and 1000 (Group 2) controls. Stroke patients and their spouses or age‐matched healthy controls will undergo extensive clinical phenotype characterization and at the participating institutes in Bangladesh. A CT/MRI scan will confirm the diagnosis of stroke, and detailed clinical and demographic data will be collected. This study will recruit participants from a single institute, and radiological findings will be confirmed by two independent experts, neurologists, and an intervention radiologist to exclude bias. Ethical clearance was obtained from the institutional review board of the National Institute of Neurosciences and Hospital (NINS&H), Bangladesh (References number IRB/NINS/2024/358). Further, informed written consent will be obtained for all subjects. Figure 1 illustrates the detailed flowchart of the MAGPIE study.

### 4.2 Sample Size and Power

We intend to enroll 3000 subjects in the study based on the current number of patients seen with radiologically confirmed strokes of 150 per month; this should be a readily achievable target within the specified time frame over 2 years. Our power estimates are based on the findings of the BRAINS study [27, 28], which demonstrated a risk ratio [RR] of 1.30. This is further enhanced by a sample allele frequency of 0.2, making it applicable to 100 candidate genes. For an allele frequency of 0.2 and an RR of 1.30, we can achieve a more stringent *p*‐value of < 0.00001 at the same 90% power. Our sub‐group analysis of different stroke types, with 1000 patients for each hemorrhagic and ischemic stroke cases and 1000 controls, enables us to identify a 1.30 RR with the allele frequency of 0.20 at a *p* = 0.01, with 80% power. The sample sizes are typically used for genome‐wide association studies with Illumina platforms. The study's strength depends on the ‘risk allele’ frequency, relative risk, and chosen type‐I and II errors. The MAGPIE study is powered to detect clinically significant gene effects among the stroke population.

### 4.3 Proband (Cases)

The MAGPIE study will recruit 2000 stroke patients, with 1000 cases each of hemorrhagic and ischemic strokes, aged 18 years or older from specialist centers in Dhaka, Bangladesh. The project has the advantage of using patients with stroke who have already been extensively investigated. Although unlike, there may be gender differences between the study groups, but these can be adjusted for minor variances. Patients suspected of stroke will be admitted to the center and assessed by a physician following national care guidelines based on international standards. In addition to clinical risk factors, we will also record exposure to environmental risk factors such as smoking, alcohol, and dietary habits. All proband will be identified using the following inclusion criteria:

1. Proband aged ≥ 18 years at the time of enrollment who will also be analyzed in subgroups to explore the genetic and phenotypic association.
2. Diagnosis of stroke using American Stroke Association (ASA) guidelines confirmed by clinical examination and CT/MRI imaging.
3. Patient or relative (unconscious or intubated patients) written informed consent.
4. Patients with any form of stroke, either mild to severe, including prior and recurrent stroke, as well as seriously ill and/or intubated stroke patients, will also be included in the study to avoid selection and reporting bias.
5. However, individuals with other significant comorbidities like neurological disorders (e.g., multiple sclerosis, brain tumors), major health comorbidities (e.g., advanced cancer), strokes secondary to other medical conditions (endocarditis, cerebral vessel aneurysm, or trauma), lack comprehensive phenotypic data, individuals on medications that could significantly influence stroke risk and skew genetic associations (e.g., experimental therapies) will be excluded from the study.

### 4.4 Controls

The control subjects will be spouses or partners, as they often come from the same geographical area and usually have similar exposure to environmental factors, especially similar lifestyles and habits. Occasionally, spouses/partners may not be available, but the MAGPIE study sample is large enough to accommodate this. The MAGPIE study will recruit 1000 controls identified by probands and will be asked to attend a hospital appointment to obtain blood samples from a direct venepuncture. If samples cannot be recruited using this method, blood collection packages will be sent to controls identified by the probands or their relatives. Controls will meet the following inclusion and exclusion criteria:

1. Should be aged ≥ 18 years at the time of enrollment.
2. No previous history of stroke, and able to provide written consent.
3. Hospitalized patients are not eligible to be controlled for the study.
4. Recruiting age and sex‐matched community volunteers will fill any lag in spouses as controls. Gender differences will be balanced using large numbers of sex‐mixed cases and/or sex stratification during genetic analysis.
5. Furthermore, this study will ensure an equal proportion of males and females in both the case and control groups to control for sex‐related differences in stroke risk. We will select controls from the same ethnic background as the cases to control for population stratification and genetic ancestry differences.
6. Controls will be from similar geographic locations and socioeconomic backgrounds to minimize environmental and lifestyle differences and ensure that controls are identical to cases in terms of medication use.

## 5 Data Collection Technique

Clinical experts will review patients' medical records before assessing enrollment eligibility. The coordinator/recruiter will interview each patient or their surrogate and controls to explain the study's purpose and their role. The proband (case) reported medical and family history will be recorded. All interview details will be documented on the patient data collection form. The following information will be recorded on the case report forms:

1. Cardiovascular risk factors: Age, sex, hypertension, diabetes, smoking status, vital signs (height, weight, waist and hip circumferences), temperature, alcohol intake (U/wk), history of prior cardiovascular disease and positive family history.
2. Biomedical data: Complete blood counts, Fasting glucose, HbA1c, fasting lipids profiles, prothrombin time, Protein C and S, fibrinogen, plasma homocysteine concentration analysis along with other biochemical markers.
3. Imaging results: A CT or MRI scan of the brain will identify the size and location of the symptomatic brain lesion. A carotid doppler duplex scan will be undertaken when patients present with anterior circulation lesions. Blood pressure and biochemistry tests will be recorded on admission results.
4. Genetic variables: Trained healthcare personnel will obtain 10 mL peripheral blood samples from stroke patients and control subjects in EDTA‐coated vials using a single venipuncture. Each sample is assigned a unique MAGPIE repository ID number and immediately stored at −80°C. Qiagen DNA isolation kits will isolate and archive high‐quality, high‐molecular weight genomic DNA from the peripheral lymphocytes. The OD260/OD280 ratio is measured and accepted as a quality control if it is above 1.8, and for lower OD260/OD280 ratios, DNA samples will be re‐purified.

### 5.1 Quality Assurance and Data Management Plan

This MAGPIE study aims to discover genetic risk factors across the entire spectrum of stroke severity while remaining unbiased. Patient confidentiality will be protected, and data will be encrypted. The MAGPIE investigators who have access to genetic data will not have access to personal details eg names, phone numbers, email and home addresses. Due to the complexity of accurately measuring individual risk profiles in polygenic disorders, subjects will not be informed of their genetic testing findings. Data collection sheets and forms will be stored confidentially at the participating centers, and research data and genetic test findings will not be recorded in the patient's clinical notes. Any future project applications from investigators to access the MAGPIE study will be assessed and strict NINS&H ethical guidelines will have to be followed. All data will be encrypted and collected during follow‐up interviews with the patient. The data will be electronically recorded and appropriate statistical tools will be used for data editing and analysis. The data and results will be presented as tables and diagrams where applicable. If data is missing randomly, the datasets will be evaluated statistically using the Litle Missing Completely At Random (MCAR) test.

### 5.2 Statistical Analysis

We will utilize SPSS v28.0, R‐4.3.2 and PLINK v2.0 software to analyse the study variables, and appropriate statistical tests will be applied to test the significance level between study groups. We will conduct separate Hardy–Weinberg equilibrium tests for cases and controls. Genotypic analyses will include allele‐dominant, recessive, and additive genetic models. Further, odds ratios will be determined using logistic regression, and the significance of association will be assessed using Chi‐square or Fisher‐exact tests, as appropriate. Data will be presented as tables, graphs, Manhattan plots, and Quantile‐Quantile (QQ) plots. A *p* < 0.05 will be considered as statistical significance when appropriate, and *p* < 10^−8^ for GWAS analysis.

## 6 Discussion

Stroke poses a significant health challenge across South Asia, especially in Bangladesh [2, 3, 4, 5] where stroke is a significant public health issue, ranking as the second leading cause of mortality and disability after heart disease. Approximately 0.22% Bangladeshi people suffer from stroke annually mainly due to an aging and increasing prevalence of risk factors like hypertension and diabetes [6, 7, 8]. Ischemic strokes account for about 75% of all cases, while hemorrhagic strokes make up approximately 15%. The high mortality rate of around 9% of all deaths, is largely due to inadequate healthcare infrastructure and lack of awareness in Bangladesh [2, 3, 7]. As in Bangladesh, the annual incidence rate in India [29, 30, 31, 32, 33] Pakistan [34, 35, 36, 37], and Srilanka [38, 39, 40] is around 150–250 per 100,000 people, and ischemic strokes are the most common, accounting for about 70%–75% of cases, while hemorrhagic strokes represent approximately 25%–30%.

Stroke accounts for 3% of the global disability burden, of which approximately 75% are ischemic, while approximately 15% are hemorrhagic, with around 12% of strokes having an uncertain diagnosis [8, 41, 42, 43, 44]. In the United States alone, there are 795,000 new and recurrent strokes reported each year, whereas South Asia, China, and Russia collectively have the highest number of stroke cases [8, 10, 45]. By 2050, an estimated 80% of the 15 million new stroke cases will occur in low and middle‐income countries like Bangladesh [44, 45, 46]. While the incidence and prevalence rates vary, common concerns include a predominance of ischemic strokes, high mortality rates, and considerable impacts on healthcare systems due to inadequate facilities and public awareness [13, 41, 45].

The MAGPIE study will be the first and largest repository of Bangladeshi stroke data, with international collaboration with the existing BRAINS consortium [27, 28]. The MAGPIE study is a dedicated genetic risk association study with well‐classified inclusion and exclusion criteria that will allow the recruitment of large numbers of stroke patients and controls to establish sufficiently powered results for risk prediction and drug discovery [47, 48]. The detailed information and DNA bank repository allow a unique opportunity to apply candidate gene and whole‐genome approaches to cerebrovascular disease. The latter aim will be achieved by increasing the availability of single‐nucleotide polymorphism (SNP) databases and gene chips. Using spouses as controls enables quick testing of candidate gene hypotheses using allelic association while controlling for environmental variability. The MAGPIE study enables the detection of both well‐known and new candidate genes, which can act independently or in conjunction with other risk genes which might help in identifying new potential therapeutic targets [47, 48, 49, 50].

### 6.1 Impact of the Study on Future Research

The creation of the first Bangladeshi stroke biobank will be an outstanding resource for future national (and international collaborative) investigators. It will motivate Bangladeshi researchers to further investigate the genetic basis of stroke in Bangladesh, potentially identifying new therapeutic targets for Bangladeshi patients. Moreover, identifying and comparing novel gene loci among stroke patients with diverse ancestries (South Asian, South Asian in the UK, and UK White British) could have far‐reaching implications on stroke medicine. This data will help devise national guidelines for treating stroke patients in Bangladesh and contribute to global stroke research allowing Bangladesh to sit at the genetics ‘top table’.

## Author Contributions

**Redoy Ranjan:** conceptualization, methodology, software, data curation, formal analysis, visualization, validation, resources, writing–original draft, writing–review and editing. **Dipannita Adhikary:** conceptualization, software, validation, formal analysis, visualization, writing–original draft, writing–review and editing. **Shanto Barman:** conceptualization, visualization, methodology, writing–original draft, writing–review and editing, resources. **Md Shuktarul Islam:** conceptualization, methodology, visualization, writing–original draft, writing–review and editing, resources. **Gie Ken‐Dror:** conceptualization, methodology, visualization, supervision, writing–original draft, writing–review and editing, resources. **Md Abdullah Yusuf:** conceptualization, methodology, data curation, validation, visualization, supervision, resources, writing–original draft, writing–review and editing. **Adneen Moureen:** conceptualization, methodology, validation, writing–original draft, visualization, writing–review and editing, resources, supervision. **Maliha Hakim:** conceptualization, methodology, data curation, validation, visualization, writing–original draft, writing–review and editing, supervision, resources. **Pankaj Sharma:** conceptualization, methodology, formal analysis, supervision, resources, visualization, validation, writing–review and editing, writing–original draft.

## Conflicts of Interest

Redoy Ranjan is an Editorial Board member of Health Science Reports but was excluded from all editorial decision‐making related to the acceptance of this article for publication. The other authors declare no conflicts of interest.

## Transparency Statement

The lead author Redoy Ranjan affirms that this manuscript is an honest, accurate, and transparent account of the study being reported; that no important aspects of the study have been omitted; and that any discrepancies from the study as planned (and, if relevant, registered) have been explained.

## Data Availability

This is an ongoing project, and data are not publicly available due to privacy or ethical restrictions. However, the data supporting this study's findings are available upon request from the corresponding author.
